# Fusion-Based Deep Learning Approach for Renal Cell Carcinoma Subtype Detection Using Multi-Phasic MRI Data

**DOI:** 10.3390/diagnostics15131636

**Published:** 2025-06-26

**Authors:** Gulhan Kilicarslan, Dilber Cetintas, Taner Tuncer, Muhammed Yildirim

**Affiliations:** 1 Department of Radiology, Elazig Fethi Sekin City Hospital, Elazığ 23280, Turkey; dr.gulhankilicarslan@gmail.com; 2 Department of Computer Engineering, Malatya Turgut Ozal University, Malatya 44210, Turkey; dilber.cetintas@ozal.edu.tr; 3 Department of Computer Engineering, Firat University, Elazığ 23100, Turkey; ttuncer@firat.edu.tr

**Keywords:** renal cell carcinoma, kidney tumor, multi-phasic MRI data, deep learning, semantic segmentation

## Abstract

**Background/Objectives**: Renal cell carcinoma (RCC) is a malignant disease that requires rapid and reliable diagnosis to determine the correct treatment protocol and to manage the disease effectively. However, the fact that the textural and morphological features obtained from medical images do not differ even among different tumor types poses a significant diagnostic challenge for radiologists. In addition, the subjective nature of visual assessments made by experts and interobserver variability may cause uncertainties in the diagnostic process. **Methods**: In this study, a deep learning-based hybrid model using multiphase magnetic resonance imaging (MRI) data is proposed to provide accurate classification of RCC subtypes and to provide a decision support mechanism to radiologists. The proposed model performs a more comprehensive analysis by combining the T2 phase obtained before the administration of contrast material with the arterial (A) and venous (V) phases recorded after the injection of contrast material. **Results**: The model performs RCC subtype classification at the end of a five-step process. These are regions of interest (ROI), preprocessing, augmentation, feature extraction, and classification. A total of 1275 MRI images from different phases were classified with SVM, and 90% accuracy was achieved. **Conclusions**: The findings reveal that the integration of multiphase MRI data and deep learning-based models can provide a significant improvement in RCC subtype classification and contribute to clinical decision support processes.

## 1. Introduction

The kidney is a basic organ that performs vital functions such as removing harmful waste and regulating body pressure. Tumor structures formed in the organ disrupt these functions and pose vital risks. Renal cell carcinoma (RCC) is among the ten most common types of cancer in adults and is the most common malignant tumor in the kidney. It constitutes approximately 2–3% of all malignant tumors globally [1,2]. In addition, according to GLOBOCAN 2020 data, more than 400,000 new kidney cancer/tumor cases have been reported worldwide, and approximately 200,000 deaths have occurred [3].

RCC is divided into three main subtypes based on histopathological features: clear cell renal cell carcinoma (ccRCC), papillary renal cell carcinoma (pRCC), and chromophobe renal cell carcinoma (chRCC). Clear cell RCC (ccRCC) is the most common type, accounting for 75% of all cases. This subtype is particularly aggressive and has a poor prognosis [4]. After clear cell carcinoma, the most common subtypes are papillary and chromophobe. Abdominal imaging is an important diagnostic tool for the detection and characterization of renal tumors. The widespread use of cross-sectional imaging techniques has led to significant progress in the detection of renal tumors. Computed tomography is the most commonly used screening method due to its short scanning time. MRI has many advantages over CT. It can be used for the initial diagnosis and staging of RCC. Radiation-free MRI provides high soft tissue contrast that visualizes the tumor’s internal structure or components. Due to soft tissue similarity, it is quite difficult to distinguish RCC subtypes with MRI alone [5]. To overcome this challenge, different MRI phases are analyzed. ccRCC lesions, due to their hyper-vascular nature, exhibit heterogeneous and intense enhancement relative to the renal parenchyma following contrast administration. They show marked enhancement in the corticomedullary phase and demonstrate washout in the nephrographic phase. In contrast, pRCC lesions display minimal, slow, and progressive enhancement, with less contrast uptake compared to renal parenchyma in the corticomedullary phase. Meanwhile, chRCC lesions, being hypo-vascular, show lower enhancement than ccRCC but greater than pRCC after contrast administration. In this study, the corticomedullary phase was denoted as “A,” and the nephrographic phase as “V”.

Although kidney biopsy is performed to distinguish malignant from benign masses, complications such as tumor cell seeding, bleeding, fistula formation, pseudoaneurysm and infection are possible along the biopsy path. The ability to distinguish tumor cells without taking tissue samples can be very valuable in facilitating diagnosis and ensuring timely treatment and can greatly assist clinicians in treatment planning [6].

### 1.1. Motivation

The use of deep learning-based models, especially convolutional neural networks (CNNs), in the classification of medical data has become widespread in the last decade. Various studies in this direction aim to provide decision support systems to radiologists. However, while the majority of current research focuses on the distinction between benign and malignant tumors and lesions, limited progress has been made in the classification of RCC subtypes [7,8]. In particular, the fact that textural and morphological features in MRI images do not differ even between different tumor types poses a significant diagnostic challenge for radiologists. Therefore, the correct classification of RCC subtypes and the determination of the treatment protocol to be applied are of critical importance. This study aims to detect RCC subtypes using MRI data from patients diagnosed with kidney cancer in different phases. Considering the insufficient number of radiologists, the development of an artificial intelligence-supported decision support system stands out as an inevitable necessity.

### 1.2. Contributions

The proposed method aims to increase diagnostic accuracy by performing feature fusion by combining multiple MRI sequences. Thus, details that cannot be detected or may be overlooked in a single image will be included in the model, providing a more reliable diagnostic process. The main contributions of this study are summarized below:

- A, T2, and V MRI phases were integrated in the evaluation process, and a comprehensive analysis was performed.
- More detailed and precise feature extraction was performed using deep convolutional neural networks (CNNs) with a dense feature set.
- Model training was performed by combining the features obtained separately from each phase, and the classification performance was evaluated in detail.
- While the literature generally focuses on Normal–Tumor distinction, this study considered Renal Cell Carcinoma as a multi-class classification and achieved high success rates.
- There are studies in the literature that generally use Computerized Tomography (CT) images and a single image. In this study, a more detailed analysis was performed by evaluating MRI images taken from different sequences. The results obtained show that the level of radiation exposure can be reduced by using MRI instead of CT.

### 1.3. Outline

The rest of the paper is organized as follows. Section 2 includes a summary of the studies in the literature. Section 3 presents the characteristics of the proposed dataset and the steps of the method in detail. Section 4 includes the evaluation of the results of the proposed method. Section 5 provides a brief evaluation of the paper.

## 2. Related Work

Advances in deep learning have significantly contributed to the increase in the number of studies on the diagnosis and classification of kidney tumors. These studies are generally divided into three main categories: classification, segmentation, and determination of the degree of the mass. The self-supervised learning method developed by Özbay et al. [7] was applied to a dataset consisting of computerized tomography (CT) images. Accuracy rates of 99.82% and 95.24% were achieved in the distinction of normal and tumor tissues, respectively. Mehemedi et al. used a deep neural network (DNN) to determine kidney tumors. In the two-stage process, UNet and SegNet were used in the segmentation, and MobileNetV2, VGG16, and InceptionV3 were used for classification [8]. Ghalib et al. classified normal and abnormal tissues by using patterns such as contrast, color, and volume [9]. Pande et al. successfully distinguished cyst, stone, and tumor using the YOLOv8 model for multiple classification using kidney CT images [10]. Zhou et al. applied the InceptionV3 model on CT images to distinguish benign–malignant kidney tumors and achieved 97% accuracy [11]. This study showed that transfer learning is more effective in kidney tumor classification. In a similar study, cyst, stone, and tumor classification based on CT images was performed using low-parameter deep learning models, and high accuracy rates were achieved [12]. Abdullah et al. proposed a deep learning-based system that allows tumors to be distinguished from stones and cysts from CT images. The proposed CNN-4 and CNN-6 models achieved 92% and 97% accuracy, respectively [13]. The most important result that draws attention in these studies is that the performance parameters of benign–malignant or cyst, stone, and all classifications are quite high.

On the other hand, a study conducted to determine tumor types focused on the distinction between clear cell renal cell carcinoma (ccRCC) and benign oncocytoma (ONC). CT images were used, and the highest accuracy rate was obtained in the EX phase, at 74% [14]. This success rate showed that distinguishing the subtypes of malignant tumors is a very difficult problem. When the current literature is examined, it is observed that classification studies are largely focused on the distinction between normal and abnormal, and that CT images are predominantly used. Studies on the distinction between different tumor types with multi-class and similar features are limited.

Sundaramoorthy et al. achieved 79% accuracy using VGG-16 and AlexNet architectures [15]. Gupta et al. used deep learning (DL) to detect renal cell carcinoma (RCC) and its subtypes (clear cell RCC (ccRCC) and non-ccRCC) from CT images. In this study, accuracy was 0.950, F1 score was 0.893, and AUC was 0.985 with CT images obtained from 196 patients [16]. Koçak et al. used ANN and SVM classifiers to classify cc-RCC, papillary cell RCC (pc-RCC), and chromophobe cell RCC (chc-RCC) kidney cancers. In their study using the TCGA dataset, the Matthews correlation coefficient (MCC) was obtained as 0.804 with SVM [17]. Uhm et al. proposed a deep learning model that can detect five major renal tumors, including both benign and malignant tumors, in CT images. The model was tested on CT images from 308 patients, and the AUC value was 0.889 [18]. Zhu et.al. proposed a neural network model to distinguish clear cell RCC, papillary RCC, chromophobe RCC, renal oncocytoma, and normal kidney tissues from pathology images. The Cancer Genome Atlas (TCGA) dataset yielded an AUC of 0.95 [19]. Han et al. proposed an image-based deep learning framework to distinguish three major subtypes of renal cell carcinoma (clear cell, papillary, and chromophobe) from CT images. In this study, in which 169 cases were examined, images were acquired in three phases. The modified Googlenet architecture achieved 85% accuracy [6]. Pan et al. investigated the prediction of Fuhrman grade in clear cell renal cell carcinoma (ccRCC) using MRI (T1- and T2-weighted imaging) and functional MRI (fMRI) modalities, including Dixon-MRI, blood oxygen level-dependent (BOLD)-MRI, and susceptibility-weighted imaging (SWI). Their study, which analyzed histopathologically confirmed cases of 89 patients, achieved an average accuracy of 85.40% using logistic regression analysis [20]. This study highlights the potential of MRI-based radiomic features, combined with qualitative radiological assessments and machine learning (ML) models, to characterize solid renal neoplasms. Further research by Said et al. utilized random forest models to analyze MRI-based quantitative radiomic features for differentiating renal cell carcinoma (RCC) from benign lesions and subtypes of RCC, such as ccRCC and papillary RCC (pRCC). Their findings demonstrated high diagnostic performance, with areas under the curve (AUC) ranging from 0.73 to 0.77 [21]. In another study, Du et al. explored the use of multiparametric magnetic resonance imaging (mpMRI) in conjunction with convolutional neural network (CNN) fusion to predict the aggressiveness of RCC preoperatively. This non-invasive approach aimed to assess tumor behavior and provided promising insights into the potential application of deep learning for tumor characterization in RCC [22]. These studies collectively underscore the growing role of advanced MRI techniques and ML-based radiomics in the accurate diagnosis, classification, and prognostication of renal neoplasms. The integrated analysis of images obtained from different MRI phases has the potential to provide greater diagnostic and prognostic accuracy. However, the number of studies exploring this approach remains limited. This highlights a significant gap in the integration of multiphase MRI imaging and its application in clinical practice, underscoring the need for further research in this area.

Different from these studies in the literature, this article focuses on the classification of clear cell, chromophobe, and papillary renal cell carcinoma (RCC) tumors on MRI images. We propose a deep learning-based method that combines the T2 phase obtained before contrast medium administration with the arterial (A) and venous (V) phases recorded after contrast medium injection. Unlike previous studies that primarily focus on individual MRI phases or rely solely on tumor regions for feature extraction, our method employs the segmentation of the entire kidney as the region of interest (ROI). This enables the model to capture both tumor-specific and organ-level structural information, enhancing its diagnostic accuracy. Furthermore, the extracted deep features from DenseNet are subsequently classified using a support vector machine (SVM), which has demonstrated superior performance in leveraging high-dimensional features compared to conventional fully connected layers.

## 3. Materials and Methods

This study aims to develop a decision support system for radiologists in order to accelerate the medical diagnosis process and to make the transition to protocol applications in a shorter time. The proposed system is designed to minimize the dependency on pathological evaluations and individual experiences of radiologists in the process of determining the types of kidney tumor. Multiple data usage is provided in order to increase the accuracy of the system in decision-making processes. By combining the features obtained from different MRI phases, the missing or skipped information in a single image is completed with the data obtained from other phases, thus reducing the error tolerance to the lowest level. The dataset definition used and the details of the method are presented in the following sections.

### 3.1. Dataset

The dataset used in this article includes 1275 MRI images obtained from 62 patients (34 male, 28 female) with definite pathological diagnoses between August 2018 and March 2025 in City Hospital. The images belong to clear cell, papillary, and chromophobe kidney cancer types and are in JPG format and have a resolution of 512 × 512. While creating medical images, T2 phases were created without drug administration. The A phase was taken at the 40th second after contrast material administration, and the V phase was taken at the 100th second after contrast material administration. The cases consist entirely of retrospective images with confirmed histopathological diagnoses. The details of the data are given in Table 1.

**Figure 1:**
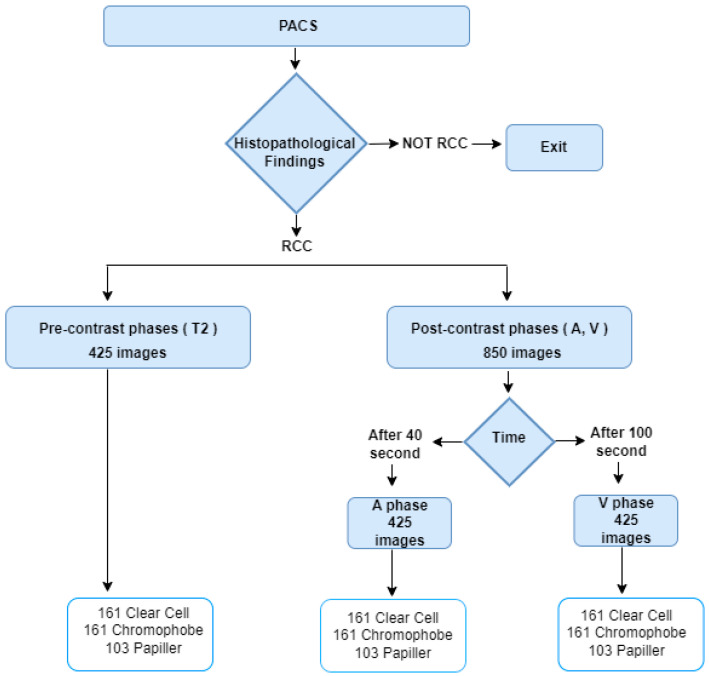
Data Curation.

The data collection procedure is given in Figure 1. For the experimental results, 80% of the data were used as training, 10% as validation, and 10% as test. Within the scope of this study, 42 of the 425 images in each phase were separated as test data.

### 3.2. Proposed Model

**Figure 2:**
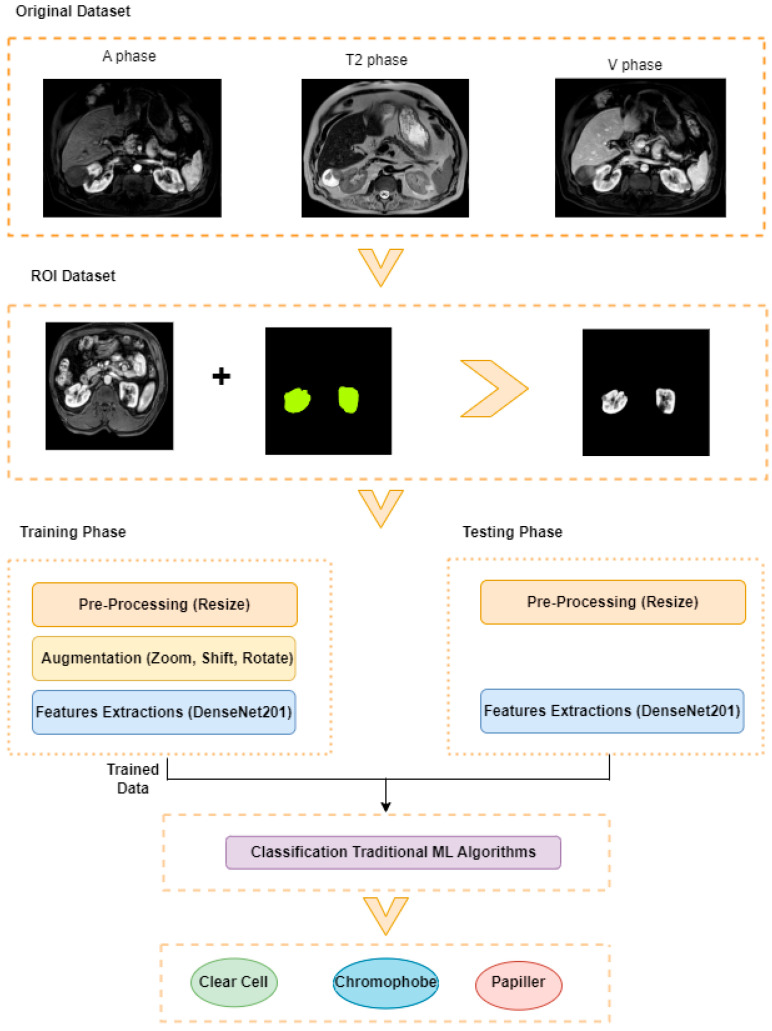
Proposed Model.

This study aims to determine the subtypes of kidney tumors using data obtained from different magnetic resonance imaging (MRI) phases. The proposed approach consists of five basic steps: Region of Interest (ROI) extraction, pre-processing, data augmentation, feature extraction, and classification. The framework of the planned model is presented in Figure 2.

#### 3.2.1. ROI Dataset

The images used in the study include different anatomical areas, such as the liver, spleen, and intestine. However, in order to process the data more quickly and provide more accurate results from the analysis, only the kidney areas (Regions of Interest, ROI) were extracted and evaluated. The Roboflow platform was used for spatial matching and segmentation of the kidneys. Polygonal ROI regions were defined by applying the semantic segmentation method, and then the transformations performed were verified by the radiologist, increasing the reliability of the system. In our study, MRI phase images were acquired at a resolution of 512 × 512 and the renal region of interest (ROI) was segmented and resized to 224 × 224 for input into the DenseNet architecture. In a similar study, Alhussaini et al. utilized 512 × 512 CT images and processed tumor-containing ROIs for their analysis; however, segmented region details were not provided [23]. Another study aimed to differentiate ccRCC from oncocytoma by extracting ROIs from T2-weighted images (T2-WI), pre-contrast T1-weighted images (T1-WI), and post-contrast arterial and venous phases. Tumor regions segmented at 100 × 100 mm were subsequently resized for input into the AlexNet model [24]. Unlike these studies, our approach involves segmenting the entire kidney, rather than solely the tumor region, to enable the deep learning model to leverage structural information for the organ as a whole.

Table 2 shows sample images containing ROI areas for different phases.

In case of image and mask matching, the Region of Interest (ROI) is determined as the kidney area whereas, in case of no match, the relevant area is classified as non-kidney. In this context, the mathematical definition of ROI is given as follows:

*Image* = *I(x,y)*

*Mask* = *C*

$$\mathrm{ROI}=\left\{\begin{array}{c} \;M\left(x,y\right)=1\;kidney\\ \;M\left(x,y\right)=0\;elsewhere \end{array}\right.$$

$$Kidney\left(x,y\right)=I\left(x,y\right).M(x,y)$$

#### 3.2.2. Pre-Processing

In many studies conducted in the literature, a great deal of emphasis has been placed on preprocessing steps, and various filtering, normalization, contrast enhancement, and denoising methods have been applied in this process. In this study, the resizing process was performed only to ensure compliance with the input dimensions of the model. Thus, the images were transferred to the model in their raw form, without any filtering or artificial intervention, and the aim was to directly learn the features in the original structure of the data. This approach allows the model to learn generalizable features over real data and prevents the loss of information that may be caused by preprocessing steps.

#### 3.2.3. Augmentation

To increase the learning capacity of the model and reduce the risk of overfitting, data augmentation techniques were applied to the region of interest data. In medical imaging, various features, particularly those linked to distinct tissue types, hold significant clinical value for diagnostic purposes. Geometric augmentation remains one of the most commonly employed techniques in this domain. In the present study, considering the diagnostic importance of texture and color, augmentation was restricted to 20% rotation, 0.2 shift, and 0.2 zoom. Geometric transformations, such as shifting images horizontally or vertically, serve as effective strategies to mitigate positional bias within the dataset [25]. The study primarily assessed the performance of dense layer architectures. In this context, the diversity of the dataset was increased by applying zoom, vertical shift, horizontal shift, and rotation operations to the images. Thanks to this method, the ability of the model to recognize different variations was improved.

#### 3.2.4. Extraction of Deep Features Using DenseNet

DenseNet is one of the pre-trained models that exhibit superior performance in various image classification tasks and is widely used in the field of deep learning [26]. In this architecture, each layer is directly connected to all subsequent layers with feedforward connections [27]. This dense connection structure facilitates the gradient flow and allows the model to be trained efficiently even on deeper structures. At the same time, thanks to this structure, learning of fine details and perception of complex visual patterns can be performed more effectively. The chained connection of inputs minimizes information loss by enabling direct access to the feature maps learned at previous levels. When pre-trained DenseNet models are fine-tuned, they can largely preserve the original and characteristic features of images [28]. Transfer learning was applied using DenseNet121, DenseNet169, and DenseNet201 models, which were initialized with pre-trained weights on the ImageNet dataset. The architecture of these models was modified by removing the final classification layers and adding GlobalAveragePooling2D and Dense layers. The pre-trained layers were frozen, allowing only the newly added layers to be fine-tuned. This approach was adopted to mitigate the risk of overfitting, particularly given the limited size of the dataset. In this study, experimental studies were carried out using DenseNet121, DenseNet169, and DenseNet201 architectures, which have a densely connected network structure, in order to effectively learn even the finest visual details.

**Figure 3:**
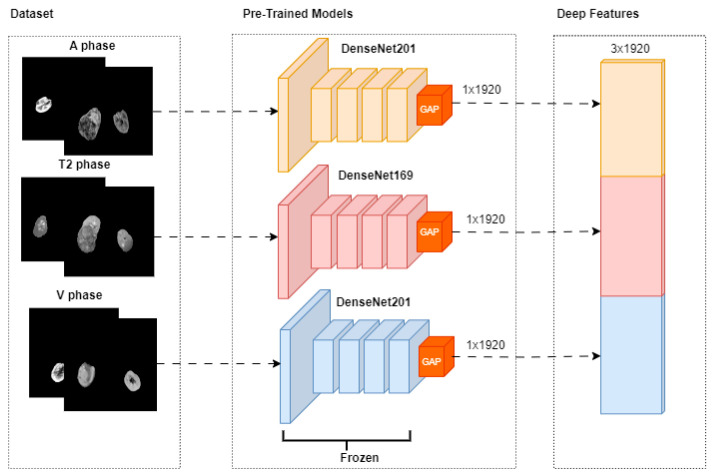
Feature Extraction.

The fusion-based pipeline utilized in this study is presented in Figure 3. The pipeline incorporates three distinct magnetic resonance imaging (MRI) phases: A-phase, T2-phase, and V-phase. Each phase is processed through pre-trained models, specifically DenseNet201, DenseNet169, and DenseNet201, respectively. Feature extraction for each phase is performed using a Global Average Pooling (GAP) layer, resulting in deep feature vectors of size 1 × 1920 for each phase. These deep feature vectors are subsequently concatenated during the fusion phase to create a unified feature representation.

$$F_{A}=GAP(DenseNet201(X_{A}))$$

$$F_{T2}=GAP(DenseNet169(X_{T2}))$$

$$F_{V}=GAP(DenseNet201(X_{V}))$$

X_A_, X_T2_, and X_V_ represent A-phase, T2-phase, and V-phase imaging data, respectively. GAP represents the Global Average Pooling process.

$$F_{fusion}=[F_{A},\;F_{T2},F_{V}]$$

$F_{fusion}$ is a 3 × 1920 combined feature vector that combines the deep features from the phases.

Ablation studies were conducted with these models on the images of each MRI phase, and the models that gave the most successful results specific to the phases were determined. Then, these successful models were combined under a hybrid structure, and the classification process was applied by performing maximum feature extraction. All experiments were performed in the T4 GPU environment using Colab Pro. The equipment was sourced from Google Colab Pro, provided by Google LLC, Mountain View, CA, USA.

#### 3.2.5. Classification

In the classification step, which is the last stage of the model, traditional machine learning methods were used to determine tumor subtypes. In this stage, the classification process was performed using previously extracted deep features with Support Vector Machines (SVM), Random Forests (RF), and K-Nearest Neighbors (KNN) algorithms. SVM transforms an input space into a high-dimensional feature space to create an ideal separation hyperplane from training examples [29]. In SVM, the input is divided into linear and nonlinear structures using margin and support vectors to create a useful decision boundary [30]. Random Forest aims to create a large number of decision trees for classification and to aggregate the results. It uses multiple DTs working together to make predictions [31]. Another algorithm used predominantly in classification is k-NN. k-NN works by finding the nearest data point or neighbor from the training dataset [32]. XGBoost is a gradient boosting framework. It aims to increase generalization ability by creating new decision trees that learn from the errors of previous models and reduce the error rate. The use of different classifiers allowed comparative analysis of the methods and made it possible to determine the algorithm that provides the most appropriate performance.

## 4. Experimental Results and Discussion

To demonstrate the effectiveness of the hybrid approach and the use of multiple data, ablation experiments were first performed. In this direction, the proposed method was compared with scenarios where only single models and single images were used. All experimental studies were conducted with fixed hyperparameter settings, and each model was trained for 50 epochs. The Adam algorithm was selected as the optimization algorithm with a mini-batch size of 16 and a learning rate of 0.001. The results of the performance parameters obtained from the confusion matrix and specified in Equations (1)–(6) for each phase are given in Table 3, Table 4 and Table 5.

(1) $$Accuracy=\frac{TP+TN}{TP+FP+TN+FN}$$

(2) $$Recall=\frac{TP}{TP+FN}\;$$

(3) $$Precision=\left(\;\frac{TP}{TP+FP}\right)$$

(4) $$F1\;Score=\left(\;\frac{2TP}{2TP+FN+FP}\right)$$

(5) $$\kappa \;=\;\frac{P_{o}\;-\;P_{e}}{1\;-\;P_{e}}$$

(6) $$\mathrm{MCC}=\frac{\left(TP\cdot TN\right)-\left(FP\cdot FN\right)}{\sqrt{\left(TP+FP\right)\left(TP+FN\right)\left(TN+FP\right)\left(TN+FN\right)}}$$

To extract detailed features from MRI phase V images, deep learning-based dense layered architectures were used. In this context, transfer learning-based DenseNet121, DenseNet169, and DenseNet201 architectures were compared, and their classification performances were evaluated. As a result of the experimental study, the highest overall accuracy rate was obtained with the DenseNet201 model. This result shows that the depth and densely connected structure of the model are effective in extracting more detailed and distinctive features from phase V images. Although low success was generally observed in the classification of papillary tumors, the DenseNet169 architecture exhibited the highest performance in distinguishing this tumor type.

In the classification experiments performed on MRI T2 phase images, similar difficulties were encountered in distinguishing the Papillary tumor class, as in phase V. However, in the classification analyses performed with T2 phase data, the highest overall accuracy rate was obtained with the DenseNet169 architecture.

According to the classification results based on MRI A phase data, DenseNet201 stands out as the most successful model when the overall accuracy and class-based F1 scores are taken into account. However, the Papillary tumor class was again the most difficult to distinguish. These findings emphasize the decisive role of the interaction between phase-based imaging and deep learning architectures on tumor classification performance. In order to demonstrate the effectiveness of the proposed model, V, T2, and A phase images were given as input to the model. Each phase image was checked by a radiologist. In the study, a total of 1275 MRI images belonging to 62 patients were labeled as clear cell, chromophobe, and papillary, three different kidney tumor subtypes, based on pathological findings. The 224 × 224 MRI phase image was given as input to the pre-trained DenseNet201 model. Deep feature vectors of fixed length 1 × 1920 were obtained for each image via the Global Average Pooling (GAP) layer at the end of each DenseNet201 model. The deep features obtained for each of the three phase images were combined and then subjected to the classification process using traditional machine learning algorithms. Performance Metrics of the Paired MRI Phase Groups are given in Table 6.

When MRI phases are evaluated in pairs, it has been observed that the results are more successful compared to single-phase evaluations. The combination of arterial and venous phases demonstrated overall effectiveness across all classes. Notably, the T2-V group exhibited the most balanced performance, with consistently high F1-scores across all subtypes. These findings highlight that combining features derived from different MRI phases leads to more robust and effective classification outcomes and promising results.

When the classification results performed with Support Vector Machines (SVMs) are examined (Table 7), it is seen that the model performs consistently well across all classes, with slight room for improvement in precision for the “Papiller” class. The high recall for “Papiller” (100%) and balanced performance across other classes indicate robustness, particularly in detecting all true cases. Overall metrics like accuracy, MCC, and Kappa above 0.84 suggest a high-quality model with reliable predictions.

**Figure 4:**
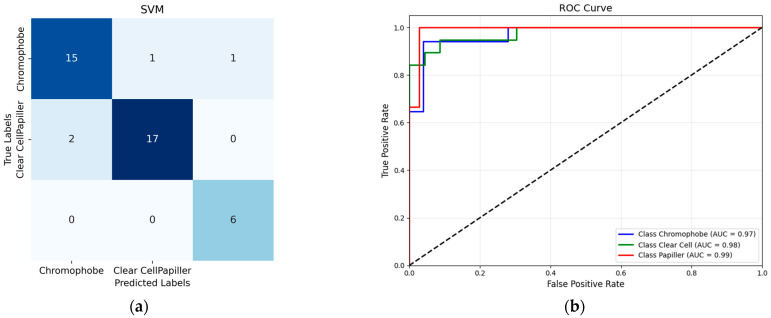
(**a**) SVM confusion matrix; (**b**) SVM ROC Curve.

According to the confusion matrix for the SVM classifier presented in Figure 4, 17 out of 19 Clear Cell tumor samples were accurately classified, while two samples were misclassified as Chromophobe tumors. Upon examining the classification performance of the Chromophobe class, it was observed that 15 out of 17 samples were correctly identified. Among the remaining two samples, one was misclassified as Clear Cell, and the other as Papillary. This misclassification shows potential visual similarities or a limited set of discriminatory features between the Chromophobe class and the other tumor classes. Notably, all samples belonging to the Papillary tumor class were correctly classified, demonstrating the robustness of the model for this class. These findings indicate that the tumor class with the highest degree of inter-class confusion under a single-method, single-dataset approach was effectively distinguished using the proposed methodology.

**Figure 5:**
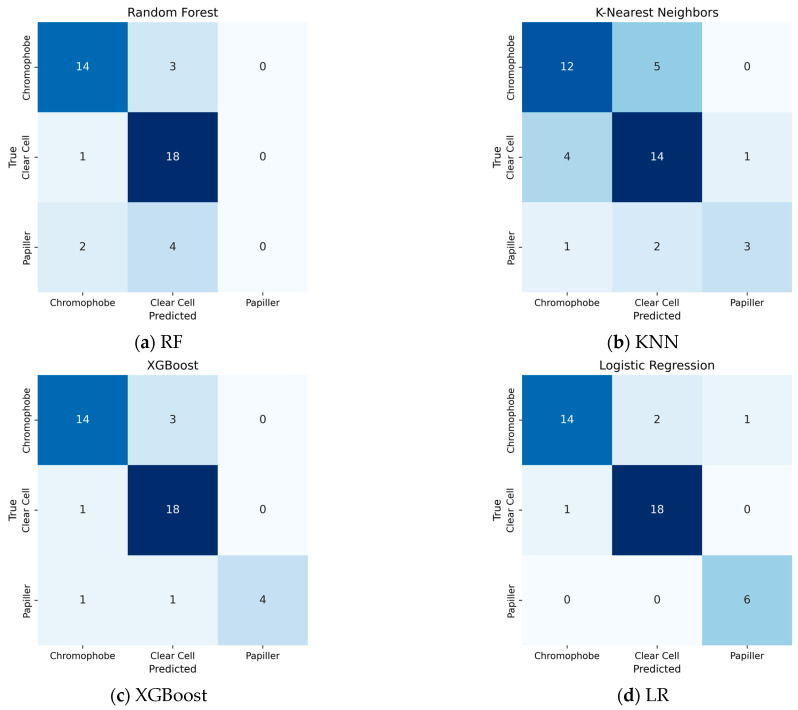
Performance of traditional ML Algorithms: (**a**) RF; (**b**) KNN; (**c**) Xgboost; (**d**) LR.

When traditional classification models other than SVM are evaluated (Figure 5), the confusion matrix obtained by the Logistic Regression algorithm is remarkable. Logistic Regression showed the most successful classification performance after the SVM model. This model managed to classify the Clear Cell tumor class with 94.7% accuracy, while this rate was 82.3% for the Chromophobe class. However, the discriminatory power of the model was significantly lower in the Papillary class, with only 66.6% accuracy. When the results of the K-Nearest Neighbor (KNN) algorithm are examined, it is observed that the model correctly classified Clear Cell tumors in 14 samples, but incorrectly classified 4 samples of Clear Cell tumors and assigned them to the Chromophobe class. This shows that Clear Cell and Chromophobe tumor classes may have structural similarities in terms of neighborhood relations that form the basis of the k-NN algorithm. On the other hand, the model was insufficient in distinguishing the Papillary class.

XGBoost produced a more successful result in the Papillary class compared to previous models, especially compared to RF. However, the confusion seen in the Chromophobe class shows that the model has difficulty in distinguishing the class in the examples belonging to this class.

RF success rate in this classification was obtained as 76%. While the Clear Cell subtype was detected with 81% accuracy, the Chromophobe subtype reached 82% accuracy. However, there were difficulties in detecting the papillary subtype.

**Figure 6:**
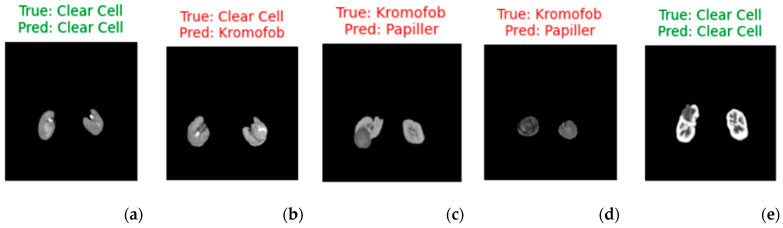
Visualized predictions with true labels (for five random selected images). (**a**,**e**) represent correct predictions, while (**b**–**d**) represent incorrect predictions.

Figure 6 shows 5 sample images classified with the hybrid method using the SVM classifier. Clear cell RCC shows more contrast than Papillary and Chromophobe RCC (Figure 6a,e), but the other two types are difficult to distinguish because they have lower and similar contrast (Figure 5c,d). Clear cell contains more cystic, necrotic, and hemorrhagic areas, while chromophobe is more solid. In Figure 5b, the lesion contains more solid areas. Therefore, the model made the wrong classification. Table 8 presents the statistical analyses.

In McNemar analysis, i → j denotes instances where class i is misclassified as class j, whereas j → i represents cases where class j is misclassified as class i. The McNemar statistic assesses whether the difference between these asymmetric misclassifications is statistically significant. In the case of the Clear Cell and Papillary classes, no misclassifications occurred between them (i.e., both i → j and j → i are zero). Consequently, the test could not be performed, indicating that the model made no errors between these two classes. For the other class pairs, no statistically significant differences were observed. This suggests that the model distinguishes between these classes with comparable accuracy and that the misclassification errors are relatively balanced. It is also important to acknowledge the potential impact of the small sample size, which may reduce the statistical power of the test and limit the ability to detect meaningful differences.

The comparison of our results with the studies conducted to distinguish kidney cancers in the literature is presented in Table 7. The most important feature that distinguishes our study from other studies is the use of MRI images. The second is the determination of kidney cancer subtypes from different phase images. The third is the determination of cancer type by combining feature vectors obtained from three-phase images. Finally, the classification success, which is considered a multi-class problem, is higher than in the literature. Table 9 shows comparisons with similar studies in the literature.

A review of the literature reveals that most existing studies predominantly utilize CT and histopathological images for renal tumor classification, while MRI—despite being a less harmful modality in terms of radiation exposure—has been comparatively underexplored. In the present study, we emphasize the diagnostic value of multiparametric MRI, demonstrating that the integration of features from different imaging phases of the same lesion can enhance classification performance. This approach allows for a more comprehensive representation of tumor heterogeneity. Furthermore, the proposed method successfully addresses the challenge of class imbalance, particularly for underrepresented subtypes, such as chromophobe and papillary RCC, by leveraging transfer learning techniques. By reducing dependence on radiologist expertise and utilizing features extracted across multiple MRI phases, the likelihood of diagnostic error is minimized. These findings suggest a meaningful advancement over previous studies that rely heavily on single-phase data or modalities associated with higher radiation risk. However, there are several limitations to our study. The first of these is the complexity of the decision-making process. Although the system architecture is complex, higher accuracy was obtained compared to ablation tests using a simpler architecture. Other challenges faced by the model in accurately classifying this subtype can be attributed to several factors. Firstly, among renal cell carcinoma (RCC) subtypes, clear cell carcinoma accounts for approximately 75% of cases, making other subtypes significantly less prevalent. This inherent imbalance in the dataset leads to a scarcity of training examples for the rarer subtypes, thereby limiting the model’s ability to learn representative features effectively. Secondly, the limited dataset size necessitates the development of techniques for effective learning from small data samples, which remains a significant challenge in deep learning. The lack of sufficient training data makes the model more susceptible to overfitting and reduces its generalizability to unseen cases. Additionally, obtaining definitive diagnoses often requires invasive, time-intensive, and costly procedures, such as biopsies or surgical interventions. These constraints not only hinder the availability of labeled data but also highlight the critical need for accurate and reliable decision-support systems.

## 5. Conclusions

This study highlights the complexity and subjectivity involved in identifying kidney tumor subtypes. The application of deep learning plays a pivotal role in accurately differentiating papillary, clear cell, and chromophobe subtypes. It is difficult to distinguish this cancer from a single MRI image due to textural similarities. In order to overcome this problem, a hybrid deep learning method is proposed in this article, where images obtained from different MRI phases are used together. Experimental findings have shown that an effective decision support system can be created by combining features obtained from different MRI phases and, thus, clinical experts can be supported in the diagnosis process. As a result of analyzing the images of each phase with densely connected deep neural networks, it was observed that the highest performance was obtained with the DenseNet201 architecture. In this direction, the images of each MRI phase were processed separately with the DenseNet201 model, and then the deep features obtained were combined to perform the classification process. This approach not only increased the representation power of the features but also minimized the error rate by compensating for information that may be missed in an image from other phase images. The results obtained support the effectiveness of the method. In addition, the reduction in the amount of ionizing radiation to which patients are exposed by using Magnetic Resonance Imaging (MRI) data instead of the frequently preferred Computerized Tomography (CT) images is another important contribution of the study. In the scope of future studies, it is aimed to process more meaningful and weighted features, instead of all features, with the integration of attention-based mechanisms. In this way, both the computational load can be reduced and the model can learn the distinctive features more effectively. Moreover the performance of the model is influenced by both hyperparameters and the scale of data augmentation. Identifying the most effective parameter configurations and augmentation strategies through optimization techniques and applying them to medical images constitutes a promising direction for future work.

## Figures and Tables

**Table 1:** Dataset properties.

	A Phase	T2 Phase	V Phase	Gender
Clear Cell	161	161	161	10 Male—12 Female
Chromophobe	103	103	103	12 Male—6 Female
Papiller	161	161	161	12 Male—10 Female
Total	425	425	425	34 Male—28 Female

**Table 2:** ROI areas in different phases.

	A	T2	V
ccRCC	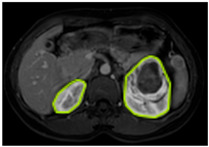	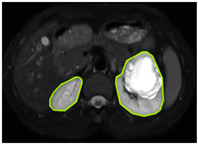	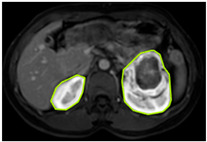
pRCC	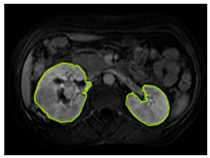	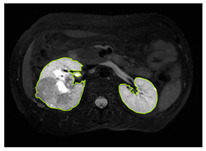	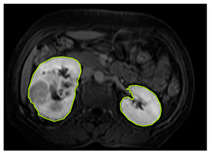
chRCC	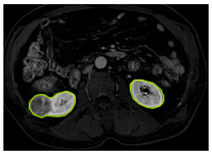	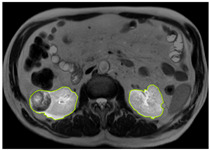	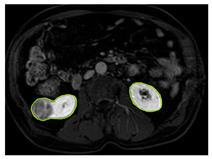

**Table 3:** Performance of V-phase data.

	DenseNet121			DenseNet169			DenseNet201		
	P	R	F_1_-Score	P	R	F_1_-Score	P	R	F_1_-Score
Clear Cell	0.75	0.60	0.67	0.77	0.50	0.61	0.81	0.65	0.72
Chromophobe	0.67	0.88	0.76	0.50	0.75	0.60	0.68	0.81	0.74
Papiller	0.20	0.17	0.18	0.80	0.67	0.73	0.43	0.50	0.46
Accuracy	0.64			0.62			0.69		

**Table 4:** Performance of T2-phase data.

	DenseNet121			DenseNet169			DenseNet201		
	P	R	F_1_-Score	P	R	F_1_-Score	P	R	F_1_-Score
Clear Cell	1.00	0.53	0.69	0.88	0.74	0.80	0.83	0.53	0.65
Chromophobe	0.59	1.00	0.74	0.78	0.82	0.80	0.60	0.88	0.71
Papiller	0.25	0.14	0.18	0.56	0.71	0.62	0.33	0.29	0.31
Accuracy	0.65			0.77			0.63		

**Table 5:** Performance of A-phase data.

	DenseNet121			DenseNet169			DenseNet201		
	P	R	F_1_-Score	P	R	F_1_-Score	P	R	F_1_-Score
Clear Cell	0.48	0.81	0.60	0.67	0.62	0.65	0.65	0.69	0.67
Chromophobe	0.58	0.44	0.50	0.54	0.88	0.67	0.64	0.88	0.77
Papiller	0.67	0.20	0.31	0.60	0.30	0.31	1.00	0.30	0.46
Accuracy	0.52			0.61			0.67		

**Table 6:** Performance Metrics of Paired MRI Phase Groups with SVM.

	A-V			A-T2			T2-V		
	P	R	F_1_-Score	P	R	F_1_-Score	P	R	F_1_-Score
Chromophobe	0.86	0.75	0.80	0.85	1.00	0.92	0.83	0.88	0.86
Clear Cell	0.86	0.90	0.88	1.00	0.79	0.88	0.94	0.89	0.92
Papiller	0.86	1.00	0.92	0.71	0.83	0.77	0.83	0.83	0.83
Accuracy	0.86			0.88			0.88		
Kappa	0.73			0.72			0.74		

**Table 7:** Hybrit CNN + SVM Performance metrics.

	Precision (%)	Recall (%)	F_1_-Score (%)
Clear Cell	88	88	88
Chromophobe	94	89	91
Papiller	85	100	92
Accuracy	90		
MCC	0.84		
Kappa	0.84		

**Table 8:** Statistical analysis.

Classes	i → j	j → i	McNemar	*p*-Value
Chromophobe vs. Clear Cell	1	2	0.0	1.0
Chromophobe vs. Papiller	1	0	0.0	1.0
Clear Cell vs. Papiller	0	0	-	0.0

**Table 9:** Literature comparison.

Ref.	Number of Classes	Dataset	Model	Parameter
Gupta et al. [16]	2	196 CT	CNN-based Resnet	A: 0.95 F_1_ score: 0.89 AUC: 0.98
Koçak et al. [17]	3	TCGA	ANN, SVM	MCC: 0.80
Ulm et al. [18]	5	308 CT	3D CNN	AUC: 0.88
Zhu et al. [19]	5	TCGA	CNN	AUC: 0.95
Han et al. [6]	3	169 BT	Modified GoogLeNet	A: 0.85
Yao et al. [33]	3	746 BT	3D convolutional neural network+fivefold cross-validation	AUC ccRCC: 0.85 AUC pRCC: 0.78 AUC chRCC: 0.79
Bai et al. [34]	4	237 CEUS	ResNet-18 and RepVGG-A0	ResNet-18 A: 0.76 RepVGG A: 0.84
Kan et al. [35]	4	4238 BT	Inception V3 ve Resnet50 +fivefold cross-validation	Inception V3 A: 0.83 Resnet50: 0.84
Chanchal et al. [36]	5	722 Hematoxylin and Eosin (H andE)	RCCGNet	A: 0.90 F1-Score: 0.89
Ye et al. [37]	2	170 US images	Vgg16 and Resnet34 using Grad-CAM	Vgg16 AUC: 0.76 Resnet34 AUC: 0.71
Proposed Method	3	1275 MRI	Densenet, Feature extraction, SVM	A: 0.90 MCC: 84 Kappa: 84

## Data Availability

Data used in this research are available upon request from the corresponding author.
